# Male gonadal dose of ionizing radiation delivered during X-ray examinations and monthly probability of pregnancy: a population-based retrospective study

**DOI:** 10.1186/1471-2458-6-55

**Published:** 2006-03-03

**Authors:** Sandra Sinno-Tellier, Jean Bouyer, Béatrice Ducot, Beatrice Geoffroy-Perez, Alfred Spira, Remy Slama

**Affiliations:** 1 INSERM, National Institute of Health and Medical Research, Unité U569 "Epidémiologie, Démographie et Sciences Sociales", IFR69, Le Kremlin-Bicêtre, France; INED, National Institute for Demographic Studies, Le Kremlin Bicêtre, France; University Paris-Sud, Faculté de Médecine, Le Kremlin Bicêtre, France; 2 Institut de Veille Sanitaire, Département Santé-Travail, Saint-Maurice, France

## Abstract

**Background:**

Male gonadal exposure to ionizing radiation may disrupt spermatogenesis, but its influence on the fecundity of couples has been rarely studied. We aimed to characterize the influence of male gonadal dose of ionizing radiation delivered during radiodiagnostic on the monthly probability of pregnancy.

**Methods:**

We recruited a random sample of women who retrospectively described 1110 periods of unprotected intercourse beginning between 1985 and 1999 and leading either to a live birth or to no pregnancy; their duration was censored after 13 months. The male partner answered a telephone questionnaire on radiodiagnostic examinations. We assigned a mean gonadal dose to each type of radiodiagnostic examination. We defined male dose for each period of unprotected intercourse as the sum of the gonadal doses of the X-ray examinations experienced between 18 years of age and the date of discontinuation of contraception. Time to pregnancy was analysed using a discrete Cox model with random effect allowing to estimate hazard ratios of pregnancy.

**Results:**

After adjustment for female factors likely to influence fecundity, there was no evidence of an association between male dose and the probability of pregnancy (test of homogeneity, p = 0.55). When compared to couples with a male gonadal dose between 0.01 and 0.20 milligrays (n = 321 periods of unprotected intercourse), couples with a gonadal dose above 10 milligrays had a hazard ratio of pregnancy of 1.44 (95% confidence interval, 0.73–2.86, n = 31).

**Conclusion:**

Our study provides no evidence of a long-term detrimental effect of male gonadal dose of ionizing radiation delivered during radiodiagnostic on the monthly probability of pregnancy during the year following discontinuation of contraceptive use. Classification errors due to the retrospective assessment of male gonadal exposure may have limited the statistical power of our study.

## Background

Studies concerning the effect of male exposure to ionizing radiation on human reproductive function have essentially focused on childhood cancer [1,2] and adverse pregnancy outcomes: stillbirths,[3,4] congenital malformations,[5,6] birth weight [7] and sex-ratio[8].

Only a few studies [9-11] have examined the effect of male exposure to ionizing radiation on the probability of pregnancy or on the occurrence of involuntary infertility. In a cohort of employees from the Nuclear Industry Family, Doyle et al. [9] did not find any association between male occupational exposure to ionizing radiation and primary infertility. Primary infertility was defined as a medical consultation for difficulties in achieving a recognized pregnancy after attempts for six months or more for couples still childless at the age of 40 years. The power of this study may have been reduced by the fact that only about half of the couples experiencing 12-month involuntary infertility decide to consult a medical doctor [12]. In a population of men serving in the French military, those who sought medical advice for a period of involuntary infertility of one year or more had more often worked in a nuclear submarine than men who had fathered a live birth with a time to pregnancy of less than 12 months[10]. However, no estimate of dose of ionizing radiation was available. In a retrospective cohort,[11] long-term survivors of a childhood or adolescent cancer, who had received radiation therapy below or above the diaphragm, had a lower fertility than their healthy siblings. No direct estimate of gonadal dose was given and fertility was also reduced for survivors who received no treatment at all.

We are unaware of any study that has yet described the potential influence of male exposure to ionizing radiation from medical X-ray examinations on fecundability, that is, the monthly probability of conceiving a clinical pregnancy among couples having unprotected intercourse.

Fecundability depends on all steps of human reproductive functions from male and female gametogenesis through to the early survival of the embryo[13]. A general mechanism by which exposure of the male gonads to ionizing radiation may influence fecundity is an alteration of sperm characteristics, leading in turn to either a decreased ability of the spermatozoa to fertilize the ovocyte or to an increased risk of early (undetected) fetal loss. A first type of effects of ionizing radiation on sperm relate to sperm concentration. A clear dose-related effect of X-rays on sperm production was shown in a population of healthy prisoners[14,15]. Azoospermia was definitively induced after testicular irradiation of 6000 milligrays (mGy, energy absorbed per kg of human tissue because of the ionizing radiation), and was temporarily induced at 300 mGy. Concentrations of both spermatozoa and spermatogonia were halved for a testicular irradiation of about 100 mGy [14]. The gonadal dose corresponding to a reduction by 50% in the number of type A spermatogonia was estimated to be about 100 mGy [14]. The fact that type A spermatogonia, the germinal cells of the testis, seem sensitive to X-ray radiation indicates a possible long-term effect of gonadal exposure to ionizing radiation. A second type of possible effects of ionizing radiation concerns the nucleus of spermatozoa. The effect of ionizing radiations on sperm DNA has been little studied among humans; in mice, ionizing radiations have been shown to affect sperm DNA [16] and to lower the development rate of the blastocysts[17]. Evidence suggests that sperm-DNA and sperm chromosomal anomalies are in the Human associated with decreased fecundability or pregnancy rates[18,19].

X-ray examinations correspond to all human body diagnostic examinations using either X or gamma ray devices, such as standard radiographs, complex vascular, urological and abdominal examinations, computed tomography (CT) scanners and nuclear medicine examinations. Some of these examinations, such as intravenous urography and abdominal CT scanner, may entail gonadal doses in the range between 10 to 100 mGy, in which an effect on sperm cells might be expected. We therefore aimed to study the association between male dose of ionizing radiation from X-ray examinations performed in adulthood and fecundability.

## Methods

### Study subjects

A cross-sectional population sample was recruited from April to July, 2000, in the Beaumont Hague canton (ward) in Normandy and in four districts near Saint-Brieuc (in Brittany), France [20]. A random list of phone numbers was drawn from the *France Télécom *telephone directory. The investigator randomly selected one woman between 18 and 60 years of age from each home to check eligibility for inclusion in the study. The woman was eligible for inclusion if she had conceived a recognized pregnancy between 1985 and 2000, was pregnant for at least three months at the time of the study, or had tried to become pregnant (involuntary infertility) for one year or more between 1985 and 2000.

Eligible women completed a standardized telephone questionnaire about medical, reproductive, contraceptive history and lifestyle factors. A telephone interview was then conducted with the current, if any, partner of the woman.

Our study was approved by the relevant ethical committees, the Commission Consultative sur le Traitement de l'Information en Matière de Recherche dans le domaine de la Santé and the Commission Nationale de l'Informatique et des Libertés. Each participant gave an oral consent before replying the questionnaire.

### Time To Pregnancy

Time To Pregnancy (TTP) was defined as the number of months of unprotected sexual intercourse from cessation of use of a method to avoid pregnancy till pregnancy or end of the attempt at pregnancy[21,22]. TTP was recorded for pregnancies that ended with a live birth, for pregnancies current for more than three months at the time of interview, and for periods of involuntary infertility lasting twelve months or more and not leading to a pregnancy whatever the outcome. This last group was included to limit the bias due to the exclusion of infertile couples usually occurring in pregnancy-based TTP studies [23-25]. We excluded couples who declared less than four sexual intercourse per month at the beginning of the period of unprotected intercourse. TTP was not defined for pregnancies that occurred while the couple was using birth control methods, even sporadically, for pregnancies following a previous pregnancy whereafter women did not use birth control methods again, and for women who had never used birth control methods[21,22]. TTP was declared by women in weeks, months and years and rounded to the nearest month. Values of zero and one month were combined and given the value of one month. We limited the study period to periods of unprotected intercourse started between January, 1^st ^1985 until July, 31^st ^1999.

### Male gonadal dose of ionizing radiation

The questionnaire completed by the man comprised three specific parts dealing with X-ray examinations carried out since 18 years of age. Dental radiographs and occupational chest X-ray examinations were not taken into account. To help recall, we asked for history of diseases potentially associated with X-ray examinations, such as a slipped disc, renal colic or cancer. We also asked the men to describe radiographs following accident, such as sprain or fracture. We then collected information on all other X-ray examinations. Finally, at the end of the questionnaire, the interviewer enumerated a list of the examinations implying the highest gonadal doses, and the man was asked if and when he had had such an examination. For each examination, the information collected was the following: radiological device (radiography, CT scanner and nuclear medicine), anatomical localization, and date (month and year). The use of contrast products, and the causes and diagnosis of the examination were also requested. We attributed a single mean dose absorbed by testes for each X-ray examination (Table 1), assuming that the dose did not vary with the calendar year. These doses stem from studies on representative samples of European patients, [26-31] and were defined in agreement with two experts in radiology.

For each period of unprotected intercourse started with the current partner of the woman, male gonadal dose was defined as the sum of the mean doses of all X-ray examinations performed from the age 18 years to the date of the end of birth control, defined as the window of exposure. Categories of male gonadal dose were defined a priori.

#### Missing data on X-ray examinations

Some examinations were incompletely described (the radiological device, the anatomical localization or date of examination was missing). The proportion of missing information for at least one of the examinations performed in the window of exposure increased as the number of examinations in the window of exposure increased. Exclusion of periods of unprotected intercourse with missing data on X-ray examinations would thus have led to preferentially discard the most exposed observations. We therefore used a single imputation approach to replace missing data on X-ray examinations[32]. The radiation dose for an X-ray examination of unknown anatomical localization, but with a known device, was assumed to be the mean value of the gonadal doses estimated for all X-ray examinations for that device in our population. For example, the testicular radiation dose for a CT scanner of unknown anatomical localization was 2.78 mGy, the mean value of the testicular doses for all CT scanners of eligible men. If the date of examination was missing, the date was drawn at random from a distribution that led to, for a given age range at the beginning of the period of unprotected intercourse, the percentage of missing dates included in the window of exposure being equal to the observed percentage of examinations with non-missing dates occurring during the window of exposure. If the radiological device was missing (9 examinations), gonadal dose for the examination was assumed to be 0 mGy.

### Statistical methods

TTP was censored at the date of medical consultation or treatment for infertility, to avoid medical intervention bias. TTP was systematically censored at 13 months of unprotected intercourse as usually done [22].

Crude probabilities of pregnancy stratified on male gonadal dose were estimated and compared using the Kaplan-Meier method and the Log-rank statistical test. These analyses were restricted to the most recent eligible period of unprotected intercourse of each woman.

We analyzed TTP using the discrete Cox model because TTP is a discrete survival duration, each menstrual cycle offering a single fertilization opportunity[33]. The link function was complementary log-log. The model included a random effect that took into account the dependence between several periods of unprotected intercourse of a given woman. The model allowed the estimation of a monthly Hazard Ratio of pregnancy (HR) comparing distinct levels of male gonadal dose. A HR greater than one corresponds to a higher estimated probability of pregnancy for the exposed group, compared to the reference group. We estimated separately the effect of male gonadal dose on the probability of pregnancy between the first four months and months 5 to 13 of the period of unprotected intercourse. This was meant to test if the association between male gonadal dose and probability of pregnancy differed between the less fecund couples (those who did not conceive in the first four months) and the more fecund couples; this is also a test of the proportional hazards hypothesis of the Cox model.

#### Confounding factors

The choice of potential confounders was based on a priori knowledge: female and male ages, calendar year at the beginning of the period of unprotected intercourse, length and regularity of menstrual period, female tobacco consumption, female and male history of urological and genital tract diseases before the beginning of the period of unprotected intercourse and study area (Normandy or Brittany) were controlled for.

We knew, for all women, whether they had ever smoked cigarettes or not. Only for periods of unprotected intercourse ending with a live birth did we have information on the number of cigarettes smoked. Smokers with a 12-month involuntary infertility period not followed by a pregnancy were assumed to have the same consumption as declared for their included livebirth, if any. Cigarettes consumption of smokers who did not conceive in the study period was assumed to be the mean consumption of women with no missing information. We asked women if they modified their tobacco consumption during the period of unprotected intercourse. If so, the women were asked to specify how long after the beginning of the period of unprotected intercourse the change occurred and what the cigarettes consumption was afterwards. This allowed to code tobacco consumption as a time-dependent variable.

Tests for trends for continuous variables transformed in categorical variables were performed using category scores. For each category, the score corresponded to the median value of the variable in the category.

Analyses were performed with Stata/SE 8.0 statistical software (Stata Corporation, College Station, TX).

## Results

### Study subjects

**Figure 1:**
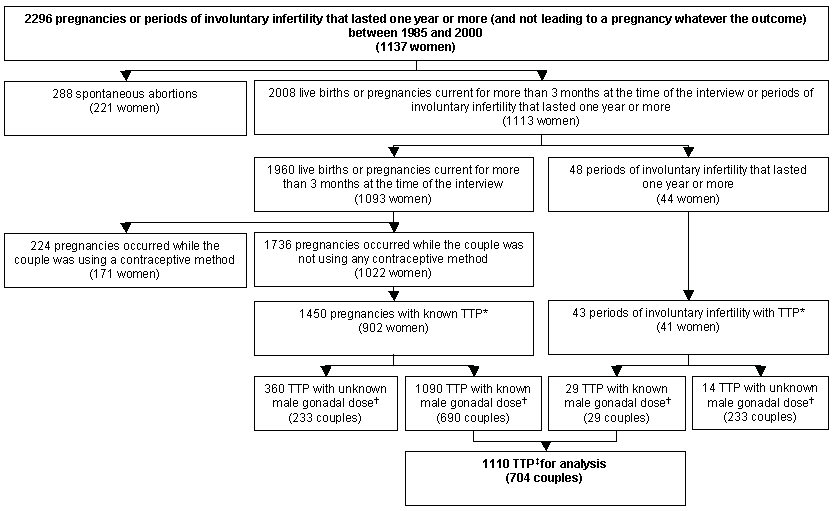
**Description of the study population**. Description of the study population, among couples living in Beaumont-Hague and in the Saint-Brieuc area, France: pregnancies that led to a live birth or a spontaneous abortion; pregnancies that occurred while the couple was using or was not using a contraceptive method. Periods of involuntary infertility lasting 12 months or more, and not leading to a pregnancy whatever the outcome. * Restricted to the periods of unprotected intercourse started between the 1^st ^of January 1985 and the 31^th ^of July 1999. ^† ^The current partner answered the questionnaire and was the partner of the woman at the beginning of the period of unprotected intercourse. Two men (3 observations) with radiotherapy for cancer before the beginning of the unprotected intercourse were excluded. ^‡ ^Eight couples (9 observations) who declared less than 4 unprotected intercourse per month were excluded.

The estimated participation rate of eligible women was 70% [20]. The 1113 eligible women reported 1960 pregnancies and 48 periods of involuntary infertility in the study period. After restriction to the observations with defined TTP and male gonadal dose, we included 704 women and their current partners, describing a total of 1110 periods of unprotected intercourse (Figure 1); these corresponded to 1068 live births, 13 pregnancies current for three months or more at the time of the interview, and 29 periods of involuntary infertility of one year or more, of which 11 occurred among couples who also had a live birth during the study period.

### Male gonadal gonadal dose of ionizing radiation

**Figure 2:**
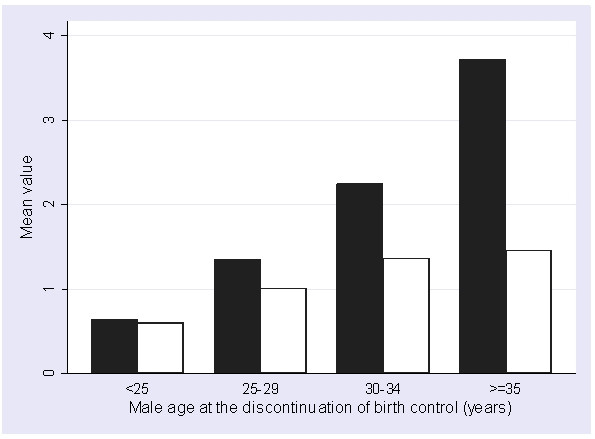
**Mean male gonadal dose and mean number of X-ray examinations experienced during the window of exposure**. Mean male gonadal dose (mGy) and mean number of X-ray examinations experienced from 18 years of age until the beginning of the period of unprotected intercourse, according to male age at the beginning of the period of unprotected intercourse (n = 1110 pregnancies and periods of involuntary infertility lasting 12 months or more, among 704 couples living in Beaumont-Hague and around Saint-Brieuc, France). ■ Male gonadal dose (mGy). □ Number of X-ray examinations.

The mean male age at the beginning of the period of unprotected intercourse was 29.2 years (25^th ^and 75^th ^percentiles, 26.2 and 31.8 years). The men had undergone between 0 and 19 X-ray examinations from the age of 18 years to the beginning of the period of unprotected intercourse (the window of exposure), with an average of 1.1 X-ray examination per man (50^th^, 75^th ^and 95^th ^percentiles, respectively 1, 2 and 4 X-ray examinations). Male gonadal dose ranged from 0 to 254 mGy (50^th^, 75^th ^and 95^th ^percentiles, respectively 0.01, 0.04 and 5.27 mGy), and was above 10 mGy for 3.0% of the observations. Radiographs of the lower limbs were the most frequent X-ray examinations (38.7%, Table 1). The number of X-ray examinations and dose increased with male age at the beginning of the period of unprotected intercourse (Figure 2).

### Monthly probability of pregnancy and male gonadal dose of ionizing radiation

The proportion of conceptions in the first month after discontinuation of birth control methods thereafter leading to a live birth was 23.5% (95% CI: 21.2–26.1); 86.8% of the couples (95% CI: 84.7–88.7) conceived within 12 months of unprotected intercourse. There was no difference in the probability of pregnancy between observations with a defined male gonadal dose (n = 1119, Figure 1) and those with no defined dose (n = 374; HR, 0,99, 95% CI, 0.80–1.23).

**Figure 3:**
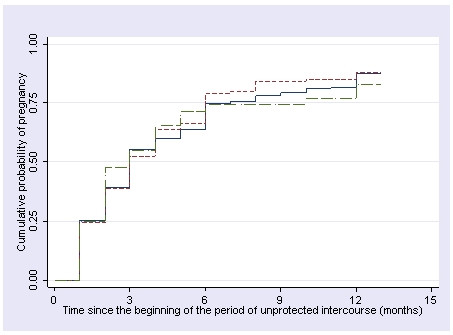
**Cumulative probability of pregnancy according to male gonadal dose**. Cumulative probability of pregnancy according to male gonadal dose (Kaplan-Meier estimates restricted to the last pregnancy or period of involuntary infertility lasting one year or more, among 704 couples living in Beaumont-Hague and in the Saint-Brieuc area, France). Male gonadal dose (mGy).  0.01–0.20 (n = 224).  0.21–5.00 (n = 124).  > 5.00 (n = 40).

Among couples with a defined dose, and when the analysis was restricted to the most recent period of unprotected intercourse for each couple, there was no difference in the cumulative probability of pregnancy according to male gonadal dose of ionizing radiation (n = 704, Logrank test, p = 0.92, Figure 3). After taking into account all periods of unprotected intercourse in a discrete Cox model (n = 1110), and taking doses from 0.01 to 0.20 mGy as a reference (n = 354 periods of unprotected intercourse), the monthly unadjusted hazard ratios (HR) of pregnancy were 0.94 (95%CI: 0.65–1.35, n = 107) for male gonadal doses between 0.21 and 2.00 mGy, 1.51 (95%CI, 0.95 to 2.41, n = 60) for doses between 2.01 and 5.00 mGy, 1.33 (95%CI: 0.68–2.61, n = 26) for doses between 5.01 and 10.00 mGy and 1.27 (95%CI: 0.67–2.44, n = 33) for doses greater than 10.00 mGy. Results changed little after adjustment for potential confounders (1023 periods of unprotected intercourse, table 2). The overall significance degree associated with male gonadal dose was 0.55.

When we split in two the period of unprotected intercourse to check the proportional hazards hypothesis, the p value for the interaction test between the two time-periods was 0.17. The adjusted HR of pregnancy, compared to the reference category, for doses between 5.01 and 10.00 mGy and for doses greater than 10.00 mGy were respectively 1.71 (95%CI: 0.83–3.56, n = 24) and 1.64 (95%CI: 0.80–3.34, n = 31) for the first four months of unprotected intercourse, and respectively 0.31 (95%CI: 0.04–2.53, n = 5) and 0.72 (95%CI: 0.17–3.08, n = 8) for the months five to thirteen.

## Discussion

Among the 1110 periods of unprotected intercourse studied, there was no clear evidence that male gonadal dose of ionizing radiation lowered the probability of pregnancy during the first thirteen months of unprotected intercourse.

The relationship between exposure to ionizing radiation and fecundability has little been studied,[11] with our study being the first on the relationship between medical X-ray examinations and fecundability. We attributed a mean gonadal dose for each type of X-ray examination and quantified gonadal dose for each man rather than dose to the whole body, less relevant biologically. Our detailed questionnaire allowed to adjust for female behavioral factors likely to influence fecundity. We included periods of involuntary infertility lasting 12 months or more; such periods are most often excluded in retrospective studies of time to pregnancy, inducing bias [25] However, the power of our study is probably limited because assessment of male gonadal dose relied on a retrospective questionnaire, which can induce classification errors.

When we split in two the period of unprotected intercourse, the point estimates of the HR of pregnancy associated with doses of 5.00 mGy or more were greater than one for the first four months of the period of unprotected intercourse (meaning an increased probability of pregnancy with male gonadal dose) and lower than one for months five to thirteen. Couples conceiving during the first four months are the most fecund ones, so that the HR of pregnancy associated with doses for this time-period reflects the effect of exposure among the most fecund couples. Symmetrically, the HR of pregnancy for the months 5 to 13 reflect the effect of exposure among the least fecund couples. Although the sample sizes were too small to draw firm conclusions, there was some evidence of gonadal doses having different effects among the least fecund couples (those not conceiving in the first four months of attempt) and the most fecund couples. If real, such a difference would have sense from a biological point of view. Variations in sperm concentrations are known to influence the probability of pregnancy only in the range between 0 and 50 millions spermatozoa/ml[34,35] Supposing schematically that men from the more fecund couples had sperm concentrations above 100 millions/ml and that the effect of ionizing radiation is to halve sperm concentration, male gonadal dose would not induce any effect on the probability of pregnancy in this group. If, on the other hand, the proportion of men with a sperm concentration lower than 50 millions/ml were high among the least fecund couples, then an effect of gonadal exposure to ionizing radiation could be expected in this group. Although not clear statistically in our study, a possibly different effect of gonadal dose between the first months of the period of unprotected intercourse and the following time period thus has some biological plausibility. Further studies are needed to confirm or rule out such a different sensitivity to doses of ionizing radiation between more fecund and less fecund couples.

One cohort, describing the influence of male exposure to ionizing radiation on the probability of pregnancy, compared subjects treated for cancer before adulthood and controls never treated for cancer[11] In this cohort, the probability of pregnancy was estimated from time between marriage and a first pregnancy. Radiotherapy and chemotherapy with alkylating agents were associated with a decreased probability of pregnancy. For patients who did not receive chemotherapy, the probability of pregnancy tended to be more strongly decreased for radiotherapy carried out below compared to above the diaphragm. The mechanisms underlying these associations are not obvious because radiotherapy below the diaphragm may more often be carried out to treat testes or other cancers associated with a decreased fecundity independently of any treatment[36] Moreover, fecundity was also reduced for survivors who received no treatment at all, compared to controls[11] Another study assessed primary infertility, defined as involuntary infertility with a medical consultation and not followed by a live birth until the age of 40 years[9] No deleterious effect of male dose of ionizing radiation was highlighted. The frequency of primary infertility was 2.5% among male workers not monitored for exposure to ionizing radiation; among monitored workers, the frequency of primary infertility varied from 2.2% for external doses between 0 and 2.49 milliSievert to 3.2% for doses above 100 milliSievert (9 cases). Occupational exposure to ionizing radiation was prospectively assessed, but primary infertility corresponds to a very severe and rare endpoint. Also, couples having involuntary infertility who did not consult a doctor for infertility were not considered as cases. These couples may represent a large proportion of couples with involuntary infertility; in a Danish study, about half of the couples with 12-month involuntary infertility had chosen to consult a medical doctor [12].

### Study subjects

The participants were not informed of the endpoints of the study until the start of the interview. The women who refused to participate (about 30% of the eligible women [20]) had similar numbers of children to those who agreed to participate (not shown). Therefore, it is unlikely that fecundity was related to the refusal of the contacted women.

Among the partners of the participating women, 74.7% of the men eligible for inclusion accepted to answer the questionnaire and provided data on gonadal dose of ionizing radiation. The probability of pregnancy and the distribution of TTP were similar for participating and non-participating men. Therefore, it is unlikely that the participation of the man was associated with the fecundity of the couple.

### Estimation of fecundity

Time to pregnancy is considered to be well recalled by women over a 15-year period in the case of pregnancy attempts leading to a live birth [21] The quality of recall of time of unprotected intercourse when the period of unprotected intercourse does not end with a pregnancy has not been studied to our knowledge. We had no information about the TTP of pregnancies that ended in miscarriages. Nonbirth outcomes like spontaneous abortions are commonly excluded from retrospective TTP studies, notably because there are doubts about the quality of recall of TTP for spontaneous abortions [21] This exclusion implies the hypothesis that X-ray radiations would have the same effect on TTP for periods of unprotected intercourse leading to a spontaneous abortion and for those leading to a live birth. The exclusion of unsuccessful attempts at pregnancy has been shown to limit the statistical power and bias TTP studies [23-25] We considered that this potential bias was of more concern than the potential bias induced by a poor quality of recall of such unsuccessful periods of unprotected intercourse, and therefore chose to include them in the analysis.

Since only a 9 to 12 months period extended from the end of the study period to the time of the interview, short TTP were over-represented among the periods of unprotected intercourse started at the end of the study period [21] This truncation might entail a bias in the case of time trends in exposure. As a check, we restricted the analysis of Table 2 to periods of unprotected intercourse started between 1985 and December 1997. The HR of pregnancy associated with a dose above 10 mGy was 1.38 (95%CI: 0.65–2.96, n = 27), very similar to the HR among the whole population, making any bias due to truncation unlikely.

Some covariates likely to influence TTP were not taken into account in the study. For example, the last contraceptive method used by the couple at the beginning of the unsuccessful attempts at pregnancy was not available.

### Pregnancy planning

Pregnancies that happened while the couple was using birth control methods have no defined time to pregnancy. These couples might have a higher fecundity than couples whose pregnancies started after discontinuation of contraception [21] Differences in male doses between these groups would result in a pregnancy planning bias [21] We estimated the magnitude of possible pregnancy planning bias in our study by assuming that pregnancies occurring while contraception was being used corresponded to very fecund couples, and we assigned a TTP of one month to all of these pregnancies. The proportion of male gonadal doses above 5 mGy was 2.7% for pregnancies occurring while the couple was using a contraceptive method, and 5.3% for the periods of unprotected intercourse taken into account in our main analysis (p = 0.17). We estimated adjusted HR of pregnancy in this new dataset including pregnancies occurring while contraception was used; the adjusted HR of pregnancy for a dose of 10.00 mGy or more was 1.17 (n = 32), versus 1.44 (n = 31) when only the eligible couples of the study were taken into account. This suggests that pregnancy planning bias may have lead to an over-estimation of the HR of pregnancy associated with male gonadal dose, which might explain why most estimated HR of pregnancy were above 1.00.

### Confounding by indication

A spurious statistical association between exposure to ionizing radiation and fecundity would be observed if X-ray examinations had been carried out as a consequence of diseases associated with decreased fecundity. This bias would correspond to confounding by indication [37] For example, percutaneous treatment with radioscopy can be used to treat varicocele, a condition that may be associated with a decreased fecundity independent of any treatment. Other examples include vas deferens opacification or a CT scanner of the abdomen or pelvis to estimate the spread of a testes cancer. None of the interviewed men reported these X-ray examinations, so confounding by indication is very unlikely in our study. Other diseases, such as vascular, neurological and endocrinal diseases, and drugs, such as neuroleptics and blood pressure drugs,[38] might indirectly influence fertility through erectile dysfunction or decreased libido. Such biases are unlikely because we excluded couples who declared a monthly sexual intercourse frequency of less than four.

### Estimation of male gonadal dose of ionizing radiation

Male gonadal exposure was defined as the sum of the estimated gonadal doses of all X-ray examination received by the man between the age of 18 and the beginning of the period of unprotected intercourse. A window of exposure starting at puberty might have been more relevant, but we believed it unrealistic to obtain information on the type and localization of X-ray examinations during teenage years in a questionnaire to the man. Moreover, X-ray radiations received in adulthood seem able to have an effect at various stages of spermatogenesis, including on germinal cells [14] We could not study the short-term effect of exposure to ionizing radiation (e.g. in the year before the beginning of the period of unprotected intercourse) because of a too small number of exposed men. X-ray examinations performed during the period of unprotected intercourse considered were excluded to guarantee that exposure precedes the health outcome studied; indeed, the longer the TTP, the higher the likelihood of an X-ray examination being performed during the period of unprotected intercourse.

Estimations of testicular dose of ionizing radiation for a given type of X-ray examination can vary by up to a factor of 10. The patient dose depends on equipment type (applied potential), radiographic technique (number of X-ray films, fluoroscopic screening time, X-ray beam projection and centering point position) and anthropomorphic characteristics[31,39] When such information is not available, classification errors in exposure occurs. In a study exploring the association between preconception paternal exposure to medical ionizing radiation and offspring birth weight, [7] exposure was classified by a dichotomous variable indicating whether the testes were likely to be in the field of the examination or not, and not by a variable with 6 categories as in our study. The question of knowing which approach leaves more room to classification bias deserves further investigation.

The type, anatomical localization and date of the X-ray examination may not always be well recalled, especially for examinations carried out a long time ago. Questions on X-ray examinations in three different sections of the questionnaire and questions on the medical history of men probably limited recall bias. We found no source of data allowing to estimate the frequency of X-ray examinations according to sex and age in France. Therefore, the magnitude of recall bias on exposure could not be directly evaluated. When we restricted the analysis to men aged 35 years or less at interview to limit the length of the recall period, adjusted HR of pregnancy associated with doses of 5.01 to 10.0 mGy and doses above 10.01 mGy were respectively 0.31 (3 observations, 95% CI, 0.04–2.50) and 0.50 (8 observations, 95% CI, 0.10–2.57), indicating that recall bias might have biased towards a lack of association the estimated effect of gonadal exposure to X-rays. Data on medical examinations in private and public health care institutes are more exhaustive since 2004–2005. This may allow, in future studies, a more precise quantification of X-ray examinations performed. A study on the quality of recall of X-ray examinations among thyroid cancer cases and controls [40] showed that, compared to medical records, some X-ray examinations were overdeclared in retrospective questionnaires whereas others were underdeclared; the associations between exposure and cancer risk were similar for both ways of assessment of exposure. Recall bias may also exist for adjustment factors, thus leaving residual confounding; the quality of recall of the frequency of sexual intercourse over a 15-year period, for instance, may be poor, as illustrated by the fact that there were 20% of missing data for this question.

Some of the men recruited in the Beaumont-Hague ward had been potentially exposed to ionizing radiation occupationally. We collected information on whether the man had ever worked in the vicinity of radioactive material or X-ray devices, or had ever carried a gamma-radiation dosimeter. The adjusted HR of pregnancy for doses of X-rays of 10.00 mGy or more was 1.32 (95%CI: 0.56–3.08, n = 19) for the 563 men potentially exposed to ionizing radiation at work, compared to 1.65 (95%CI: 0.54–5.07, n = 12) for the 460 unexposed men, giving no evidence that our estimate was strongly biased by potential occupational exposure.

#### Treatment of missing data on X-ray examinations

For 209 periods of unprotected intercourse (19% of the observations), either the date or the anatomical localization was missing for one of the X-ray examinations. The missing information for these examinations were imputed by using a single imputation approach[32] Excluding these missing values would have meant excluding the corresponding periods of unprotected intercourse. This could have resulted in a bias. When these 209 periods of unprotected intercourse were excluded, the adjusted HR of pregnancy for male doses of 10.00 mGy or more was 1.71 (95%CI: 0.78–3.74, n = 25), compared to 1.44 (95%CI: 0.73–2.86, n = 31) when the missing data was imputed.

Assuming no bias, the probability to detect a decrease in fecundability by 50% for men exposed to a gonadal dose above 10 mGy was about 70% for a significance level of 0.05 [22].

## Conclusion

Overall, our results do not support the hypothesis of a chronic deleterious effect of male gonadal exposure to medical X-ray examinations on the monthly probability of pregnancy in the year following discontinuation of birth control; this finding must be interpreted with caution because recall errors on exposure and pregnancy planning bias were present and were both in the direction of an under-estimation of a deleterious effect of exposure, if any. There was a suggestion that the association between male gonadal dose and probability of pregnancy could differ between the most fecund couples and those who failed to conceive in the four first months of unprotected intercourse.

## List of abbreviations

CI: Confidence Interval

CT: Computed tomography

HR: Hazard Ratio

mGy: milliGray

TTP: Time To Pregnancy

## Competing interests

The author(s) declare that they have no competing interests.

## Authors' contributions

AS, RS and BD conceived the study and carried out the data collection. SST, BGP and RS defined and implemented the approach used to assess exposure to ionizing radiation (the participation of Valérie Briand at this step is acknowledged). SST and RS performed the statistical analysis and drafted the manuscript. JB, BD, BGP and AS reviewed the manuscript and made significant comments. All authors read and approved the final manuscript.

## Pre-publication history

The pre-publication history for this paper can be accessed here:



## Figures and Tables

**Table 1:** Diagnostic X-ray examinations experienced by men during the window of exposure*.

Type and anatomical localization of X-ray examinations	Mean gonadal dose per examination (mGy)	Examinations performed from 18 years to the beginning of the period of unprotected intercourse	
		
		Number of X-ray examinations	%
**Simple radiographs**			
Skull, head and neck	0.02	79	6.3
Upper limbs	0.01	202	16.1
Chest, lungs and thorax	0.02	51	4.1
Upper lumbar spine	0.1	28	2.2
Lower lumbar spine	0.6	127	10.1
Abdomen	0.7	10	0.8
Pelvis and hips	4	71	5.6
Lower limbs	0.01	487	38.7
*Unknown localization*	0.42^†^	77	6.1
			
**Computed Tomography scanners**			
Skull, head and neck	0.10	16	1.3
Upper limbs	0.05	2	0.2
Chest, lungs and thorax	0.02	2	0.2
Upper lumbar spine	0.45	2	0.2
Lower lumbar spine	3.1	13	1.0
Abdomen	3.7	4	0.3
Pelvis and hips	20	7	0.6
Lower limbs	0.05	7	0.6
*Unknown localization*	2.78^†^	1	0.1
			
**Nuclear medicine examinations**			
Skull	1.00	2	0.2
Body scintigraphy	3.00	1	0.1
			
**Complex examinations**			
Thoracic angiography	1.5	7	0.6
Lower limbs angiography	5.1	1	0.1
Phlebography below diaphragm	5.5	3	0.2
Upper limbs arthrography	0.05	0	0.0
Lower limbs arthrography	0.10	1	0.1
Myelography	2.2	2	0.2
Barium meal	3.7	21	1.7
Intravenous urography	50	22	1.8
Cystography	20	2	0.2
			
***Unknown device***	0	9	0.7
			
**Total**	-	1257	100

(n = 1110 pregnancies or periods of involuntary infertility lasting one year or more among 704 couples living in Beaumont-Hague and in the Saint-Brieuc area, France).

* The window of exposure spans from the age of 18 years until the beginning of the period of unprotected intercourse. The dose estimated to be delivered to the gonads (mGy) for each type of examination is also given.

^† ^Mean value of the gonadal doses assessed for all X-rays examinations for that device, whatever their localization.

**Table 2:** Adjusted Hazard Ratios of pregnancy and 95% confidence intervals according to male gonadal dose.

	n	%	HR *	95% CI		*p values*
**Male gonadal dose (mGy)**						*0.55*
No X-ray examination	491	48.0	1.03	0.80	1.31	
0.01–0.20	321	31.4	1			*p for trend*
0.21–2.00	101	9.9	0.86	0.59	1.26	*0.27*
2.01–5.00	55	5.4	1.32	0.81	2.15	
5.01–10.00	24	2.3	1.38	0.68	2.80	
> 10.00	31	3.0	1.44	0.73	2.86	

**Female age ^† ^(years)**						*0.15*
< 20	21	2.1	0.57	0.28	1.17	
20–24	321	31.4	1.00	0.79	1.28	*p for trend*
25–29	466	45.5	1			*0.05*
30–34	177	17.3	0.79	0.59	1.07	
35–39	34	3.3	0.88	0.45	1.71	
≥40	4	0.4	0.19	0.04	1.00	

**Menstrual cycle regularity**						*0.09*
Regular	708	69.2	1			
Irregular	159	15.5	0.69	0.46	1.03	
Oral contraception as usual birth control	156	15.3	1.23	0.86	1.78	

**Menstrual cycle length (days)**						*0.07*
< 21	16	1.6	0.91	0.36	2.29	
21–27	255	24.9	1.24	0.90	1.70	
28	433	42.4	1			
29–35	251	24.6	0.92	0.66	1.27	
> 35	68	6.6	0.51	0.27	0.95	

**Female tobacco consumption ^‡ ^(cigarettes/day)**						*0.24*
0	639	62.5	1			
1–10	244	23.8	0.92	0.71	1.20	*p for trend*
11–20	129	12.6	0.86	0.61	1.20	*0.08*
> 20	11	1.1	0.36	0.13	1.02	

**Female genital and urinary tract diseases ^†,§^**						*0.16*
No	939	91.8	1			
Yes	84	8.2	0.75	0.50	1.12	

**Male age ^† ^(years)**						*0.42*
< 25	162	15.8	0.74	0.51	1.09	
25–29	466	45.6	0.94	0.74	1.21	*p for trend*
30–34	288	28.2	1			*0.36*
35–39	87	8.5	0.81	0.53	1.24	
≥ 40	20	1.9	0.68	0.30	1.54	

**Male genital and urinary tract diseases**^†,¶^						*0.80*
No	888	86.8	1			
Yes	135	13.2	0.95	0.68	1.33	

(n = 1023 pregnancies or periods of involuntary infertility lasting one year or more for 657 couples living in Beaumont-Hague and in the Saint-Brieuc area, France).

* Hazard Ratio (HR) of pregnancy adjusted for calendar year at the beginning of the period of unprotected intercourse, area of inclusion and for the variables detailed in the table. A value below 1 indicates a decreased probability of pregnancy compared to the reference category.

^† ^At the beginning of the period of unprotected intercourse.

^‡ ^During the period of unprotected intercourse (time-dependent variable). Sample sizes are given for the first month of the period of unprotected intercourse.

^§ ^Previous history of ovarian or uterine cancer, salpingitis, endometriosis, ovarian cysts or infection, genital tract operation, Chlamydia infection.

^¶ ^Previous history of cancer, mumps with testes injury, cryptorchidism, inguinal hernia, varicocele, testes torsion, gonococci, epididymitis, cystis, prostates, genital tract operation, Chlamydia infection.
